# Synergistic anti-malarial action of cryptolepine and artemisinins

**DOI:** 10.1186/s12936-016-1137-5

**Published:** 2016-02-16

**Authors:** Arnold D. Forkuo, Charles Ansah, Kwesi M. Boadu, Johnson N. Boampong, Elvis O. Ameyaw, Ben A. Gyan, Andrea T. Arku, Michael F. Ofori

**Affiliations:** Department of Pharmacology, Faculty of Pharmacy and Pharmaceutical Sciences, College of Health Sciences, Kwame Nkrumah University of Science and Technology, Kumasi, Ghana; Department of Biomedical and Forensic Sciences, School of Biological Science, College of Agriculture and Natural Sciences, University of Cape Coast, Cape Coast, Ghana; Department of Immunology, Noguchi Memorial Institute for Biomedical Research, University of Ghana, Legon, Ghana

**Keywords:** *Cryptolepis sanguinolenta*, Cryptolepine, Malaria, Anti-malarial drug combinations, Artemisinins, Synergy

## Abstract

**Background:**

Cryptolepine (CPE) is the major indoloquinoline isolated from the popular West African anti-malarial plant, *Cryptolepis sanguinolenta.* CPE possesses various pharmacological activities with potent anti-malarial activity against both chloroquine (CQ)-resistant and -sensitive strains. The search for safe and novel anti-malarial agents and combinations to delay resistance development to *Plasmodium falciparum* directed this work aimed at evaluating the anti-malarial interaction and safety of CPE in combination with some artemisinin derivatives.

**Methods:**

The in vitro SYBR Green I, fluorescent-based, drug sensitivity assay using a fixed ratio method was carried out on the CQ-sensitive plasmodial strain 3D7 to develop isobolograms from three CPE-based combinations with some artemisinin derivatives. CPE and artesunate (ART) combinations were also evaluated using the Rane’s test in ICR mice infected with *Plasmodium berghei* NK-65 strains in a fixed ratio combination (1:1) and fractions of their ED_50_s in order to determine the experimental ED_50_ (Z_exp_) of the co-administered compounds. Isobolograms were constructed to compare the Z_exp_ to the Z_add_.

**Results:**

CPE exhibited promising synergistic interactions in vitro with ART, artemether and dihydroartemisinin. In vivo, CPE combination with ART again showed synergy as the Zexp was 1.02 ± 0.02, which was significantly less than the Z_add_ of 8.3 ± 0.31. The haematological, biochemical, organ/body weight ratio and histopathology indices in the rats treated with CPE at all doses (25, 50, 100 mg kg^−1^*po*) and in combination with ART (4 mg kg^−1^) showed no significant difference compared to the control group.

**Conclusion:**

The combination of CPE with the artemisinin derivatives were safe in the rodent model and showed a synergistic anti-malarial activity in vivo and in vitro. This study supports the basis for the selection of CPE as a prospective lead compound as the search for new anti-malarial combinations continues.

## Background

Malaria remains one of the world’s leading cause of childhood morbidity and mortality and accounts for 10 % of childhood deaths in sub-Saharan Africa [1]. In 2015, 214 million new cases occurred worldwide with the African region accounting for most cases (88 %) and mortality (90 %) from the disease [1]. With the development of artemisinin resistance and the delayed parasite clearance with the ACT in the Greater Mekong sub-region (GMS) [2], the search for safe and selective chemotherapeutic agents with efficacy that will not be compromised by *Plasmodium* remains the focus. The problem of resistance of *Plasmodium* to anti-malarials in the high endemic regions has left these regions with high incidence of treatment failure considering the few and affordable treatment options available [3].

**Fig. 1:**
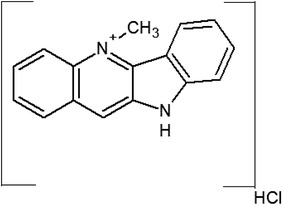
Cryptolepine hydrochloride

The contribution of medicinal plants to the development of novel anti-malarial combinations cannot be underestimated since most of the anti-malarial drugs in use are either obtained directly from plants or developed using lead structures from plants [4]. The limited availability coupled with the high cost of pharmaceuticals in many African countries has resulted in the majority of the populace depending on herbal medicines for treatment of several ailments, including malaria [5]. The aqueous root extract of *Cryptolepis sanguinolenta* is a well-known anti-malarial agent in West African ethnomedicine. It has gained popularity among indigenes for decades and is now packaged for use in hospitals and other herbal centres. Bioactive compounds from medicinal plants used traditionally is an important approach for identifying novel and potent anti-malarial drug candidates. Cryptolepine (Fig. 1) (CPE) is the major indoloquinoline alkaloid isolated from *C. sanguinolenta.* CPE is reported to possess several biological activities, including antihyperglycaemic [6], antifungal [7], antihypertensive [8], antibacterial [9], anti-inflammatory [10], antiplasmodial activities [11–13].

CPE has been studied extensively and has been found to be an important lead compound in the search for effective and novel anti-malarial drugs. The aqueous root extract of *C. sanguinolenta* is patronized in rural West Africa as a herbal extract in the treatment of malaria even for patients who are on prescribed artemisinin derivatives. A possible interaction of the herbal extract with widely used artemisinin derivatives is not known. The present study aimed at determining the possible toxicity and anti-malarial interaction (in vitro and in vivo) when CPE, the major alkaloid of the plant is combined with some artemisinin derivatives. The outcome of the study is expected to provide information on the safety, activity and possible interaction when CPE is combined with these standard anti-malarial agents.

## Methods

### Materials

CPE hydrochloride (purity 98.9 %) was isolated from *C. sanguinolenta* as described by Kuntworbe *et al.* [14]. Briefly, the isolation of the compound proceeded through the exhaustive extraction of the root powder of *C. sanguinolenta* by soxhlet with methanol, followed by a combination of liquid–liquid extraction and column chromatography leading to the isolation of the pure compound in high yield. The isolated CPE was identified by the mass spectrometry, thin layer chromatography (TLC), High Performance Liquid chromatography (HPLC) and the melting point determination. Artemether (ARM) and dihydroartemisinin (DHA) (Novartis Pharma AG, Basel, Switzerland), artesunate (ART) and ethanol (70 %) were obtained from Sigma-Aldrich (St Louis, MO, USA). Gentamicin was obtained from Invitrogen Life Technologies Inc. (Carlsbad, CA, USA). RPMI-1640 medium, streptomycin/penicillin, l-glutamine and HEPES were obtained from Gibco BRL Life Technologies (Grand Island, NY, USA).

### Animals

Healthy Sprague-Dawley rats (150-250 g) and mice (ICR) infected with *Plasmodium berghei* NK-65 (chloroquine (CQ)-sensitive strain) (25-30 g) were purchased from the Noguchi Memorial Institute for Medical Research, University of Ghana, Legon, and kept in the animal house of the Department of Pharmacology, Kwame Nkrumah University of Science and Technology (KNUST), Kumasi, Ghana. The malaria parasites in the mice were kept alive by continuous intraperitoneal passage to healthy mice every 4 days [15]. These infected mice were used for the antiplasmodial study.

All animals were housed under constant environmental conditions (21 ± 2 °C, 40 ± 5 % humidity, and 12-h light-dark cycles) and were allowed free access to food and water. The rodents were housed in stainless cages (35 × 48 × 18 cm) with soft wood shavings as bedding and allowed free access to water and commercial feed (Agricare, Tanoso, Kumasi, Ghana). All animals were fasted overnight (12 h) before oral dosing but food was returned to cages 4 h post oral dose. All animals used were naïve and used only once. The use of animals was in agreement with the National Institute of Health Guidelines for Care and Use of Laboratory Animals (1985) and was approved by the Ethical Review Committee of the Faculty of Pharmacy and Pharmaceutical Sciences, KNUST, Ghana (PHARM/ETHIC/ET173/15).

### In vitro malaria parasite cultivation

The asexual intra-erythrocytic stage of *Plasmodium falciparum* laboratory strain 3D7 was continuously cultured in vitro based on the approach described by Trager and Jensen [16] with slight modifications. The parasites were cultured in erythrocytes (sickling negative; O rhesus positive) fortified in complete culture medium (pH 7.3). The complete culture medium consisted of filter-sterilized RPMI 1640 solution supplemented with 0.5 % AlbuMAX II and hypoxanthine (0.04 %) and buffered with 0.4 % sodium bicarbonate (NaHCO_3_) and 0.72 % HEPES (N-2-hydroxyethylpiperazine-N-2-ethanesulfonic acid). Gentamicin (0.005 mg/mL) was added to the final solution.

The parasites were grown in culture flasks incubated under a gas phase of 92 % N_2_, 5 % CO_2_ and 3 % O_2_ at 37 °C. The cultures were maintained daily by changing the media and monitoring parasite viability and growth by light microscopy. Parasitaemia levels in the cultures were kept between 2 and 8 %, with 5 % haematocrit.

### In vitro anti-malarial interaction assay

The in vitro anti-malarial activity of CPE and in combination with the artemisinin derivatives against *P. falciparum* (3D7, CQ sensitive) was investigated using the SYBR Green I-based fluorescence assay [17]. d-Sorbitol treatment (5 %) was used to generate parasite cultures with high synchronous ring stage for each assay.

Stock solutions of drugs were prepared in 70 % ethanol. CPE, ART, ARM, and DHA stocks were prepared at 1 mM. The concentration ranges of CPE were between 32.5 and 2080 nM, those for ART were between 0.5 and 32 nM, and those for ARM and DHA were between 0.625 and 48 nM. Previously described fixed ratio interaction assay was employed [18] in the investigation of the combined effects of CPE with these artemisinin derivatives. A volume of 10 µL of four fixed drug ratios (4:1, 3:2, 2:3, 1:4) of CPE (Drug A) with an artemisinin drug (Drug B) were prepared in each of the assay medium. This was followed by twofold serial dilutions of each well and ensuring that the IC_50_ of each drug alone (5:0 and 0:5) falls approximately at the mid-point of the serial dilution. Each culture well was further seeded with 90 µL of parasite culture to obtain seven desired final concentrations for each combination assay. The final haematocrit of cultures were adjusted to 2 % by dilution with complete parasite medium (CPM) and parasitaemia was stepped down to 1 % with washed uninfected red blood cells (RBCs). The plates were arranged in a clean modular incubation chamber (Billups-Rothenberg Inc, USA) and flushed with mixed gas (gas contains 92 % N_2_, 5 % CO_2_, 3 % O_2_) for 5 min. The chamber with the assay plates was placed in an incubator for 48 h and set at 37 °C. After the 48-h incubation, the plates were wrapped in aluminium foil and stored at −30 °C overnight.

The plates were thawed and thoroughly mixed with 100 μL malaria SYBR Green 1 fluorescent (MSF) lysis buffer containing SYBR Green. The plates were incubated at room temperature in the dark for an hour and fluorescence data were acquired using fluorescence multi-well plate reader (Tecan Infinite M200 Pro) with emission and excitation wavelength at 535 and 485 nm, respectively. The experiment was done in triplicate. The plates were examined for the RFU per well. The fluorescence of the non-parasitized RBCs and the background fluorescence of the empty well were each subtracted from the fluorescence readings. The resultant fluorescence were converted to percentages and plotted against the log of the drug concentration. The data obtained was analysed using GraphPad Prism (GraphPad 6 Software, San Diego, CA, USA) by non-linear regression (sigmoidal dose-response/variable slope equation) to yield the IC_50_ (50 % inhibitory concentration) which served as a measure of the anti-malarial activity.

### In vivo anti-malarial interaction assay

The ED_50_ of CPE was estimated using the curative (Rane) test as described by [19]. In this test, schizonticidal activity of CPE was determined in established infection. On day 0, Giemsa-stained thin blood smear of the donor mice were prepared to determine the RBC count and percentage parasitaemia by using an improved Neubaur Counting Chamber. Blood from donor mice was obtained by cardiac puncture. Physiological saline was used in diluting the collected blood. Each mouse was intraperitoneally injected with 0.2 ml of 1 × 10^6^*P.berghei* NK-65-infected erythrocytes on the first day (day 0).

After 72 h, and following confirmation of parasitaemia, the mice were divided into five groups of five mice per group. These groups were treated with CPE at doses of 3, 10, 30, and 100 mg kg^−1^. The positive control group was treated with ART (1, 3, 10 and 30 mg kg^−1^) and 10 ml kg^−1^ of physiological saline was given to the negative control group. The drug treatment lasted 5 days at a single daily dosing. Blood smears were collected daily before each drug administration and monitored for the parasitaemia level under the microscope.

To obtain the combination potency of the co-administered CPE and ART, the two agents will be assayed for anti-malarial activity as describe earlier at doses of their respective ED_50_’s (equi-effective doses) and in fixed ratio combinations (1:1) of fractions of their respective ED_50_ values of ^1^/_2_, ^1^/_4_, ^1^/_8_, ^1^/_16_ [20, 21]. The ED_50_ for the combination (Zexp) will also be determined as described earlier. To determine the type of interaction exhibited by the co-administration, an isobologram consisting of CPE on the abscissa and ART on the ordinate will be constructed. The Zexp will be plotted and compared to the Zadd statistically using‘t’ test. Synergistic anti-malarial effect will be achieved when the Zexp is higher (ED_50_ significantly lower) than the Zadd. If the ED_50_’s are not statistically different, the effect of the combination is additive. Antagonistic anti-malarial effect will be observed when the Zexp is significantly higher than the Zadd.

On day 9 post malaria infection, all the mice from each co-administered group were sacrificed and blood collected by cardiac puncture for haematological analysis. The stomach, liver and kidneys were also harvested for histopathological analysis.

### Safety evaluation of CPE and ART in healthy rats

Healthy male Sprague-Dawley rats (150–250 g) were divided into eight groups of five animals in each group. The groups received 25, 50 and 100 mg kg^−1^ body weight of CPE orally, daily for a period of 3 days. Another set of groups received a concurrent administration of CPE 25, 50 and 100 mg kg^−1^ body weight with ART (4 mg kg^−1^) orally for 3 days. A group of five animals each also received 4 mg kg^−1^ of ART orally, daily for a 3-day period. The control group received equal volume of physiological saline solution for the period of the experiment. Each group was then closely observed for signs of toxicity.

On the third day, the rats were sacrificed by cervical dislocation, the jugular vein cut and blood allowed to flow freely into tubes with and without ethylenediaminetetraacetic acid (EDTA) as coagulant. Haematological and biochemical parameters were determined by an automatic analyser (Sysmex XT-2000 L CELL-DYN 1700, Abbot Diagnostics Division, Abbot Laboratories, Abbot Park, IL, USA) and automated analyser ATAC 8000 Random Access Chemistry System (Elan Diagnostics, Smithfield, RI, USA), respectively. Selected organs including the spleen, liver, kidneys, and stomach were excised, trimmed of fat and connective tissue, blotted dry and weighed on a balance. The relative weights of the organs were calculated and expressed as per cent of body weight.

### Toxicological assessment of CPE and ART co-administration in the Rane’s test

Sections of the tissue from the stomach, kidney, spleen, and liver of both healthy rats and *P. berghei*-infected mice were used for histopathological examination. Samples collected were washed separately in physiological saline and fixed in formalin (buffered with 10 % NaH_2_PO_4_) for 24 h and used for the histology study. Previously described method reported by [22] was used in the processing of the tissues. Tissue sections of each target organ were fixed in Bouin’s solution for 12 h and embedded in paraffin. For histopathological examination, tissues sections (5 µm) were rehydrated, stained with haematoxylin and eosin (H and E) and observed under light microscope.

## Data analysis

### In vitro anti-malarial interaction assay

Growth inhibition due to CPE and the other anti-malarial agents defined as the difference between the percentage parasitaemia of each treatment group and the corresponding control was calculated. The FIC_50_s for each fixed dose ratio were calculated from the IC_50_ values obtained from the dose-response curves. ΣFIC_50_s of CPE with the artemisinin derivatives were represented as isobolograms calculated using the equation:

$$\begin{aligned} \Sigma {\text{FIC}}_{{50}}& = \left( {\frac{{IC_{{50}}\,{\text{ of cryptolepine in combination}}}}{{IC_{{50}} \,{\text{ of Cryptolepine alone}}}}} \right) \hfill \\ &\quad+ \left( {\frac{{IC_{{50}}\, {\text{ of Drug B in combination}}}}{{IC_{{50}}\, {\text{ of Drug B alone}}}}} \right) \end{aligned}$$

The FIC_50_ of CPE was plotted against each of the anti-malarial agent to obtain isobolograms representing each of the four drug ratios. Convex curves denote antagonism; straight lines denote additivity and concave curves denotes synergy [18]. The nature of the interaction was explained using the following ΣFIC_50_ values: ΣFIC_50_ <0.8 indicates synergism, ΣFIC_50_ 0.8–1.4 indicates additive, ΣFIC_50_ ≥1.4 indicates antagonism [23, 24]. The overall nature of the anti-malarial interaction was based on the mean ΣFIC_50_s.

### In vivo anti-malarial interaction assay

The antiplasmodial activity was determined using the equation:

$$\begin{aligned}&{\text{\% Suppression}} \\ &\,\,= \frac{{ {\text{Parasitaemia of negative control}} - {\text{Parasitaemia of test }}}}{{ {\text{Parasitaemia of negative Control}}}} \times 100 \end{aligned}$$

The potency of CPE and ART were estimated from their log-dose response curves. An isobologram consisting of an additivity line that connects ED_50_, Drug A on the vertical axis to ED_50_, Drug B on the horizontal axis was plotted. The estimated potencies (ED_50_s) of CPE and ART in both tests were also used to compute the theoretical potency (Zadd) as follows:

$$Zadd = f(ED_{{50}})\,of\, artesunate + (1 - f) ED_{{50}}\, of \,cryptolepine$$

where *f* is the fraction of the each component in the mixture.

For the combination assay, mid points (ED_50_’s) will be determined using nonlinear regression (three-parameter logistic) equation: The fitted midpoints of the curves (Zexp and Zadd) will be compared statistically using *t* test.

Data for toxicity studies were presented as Mean ± SEM. The GraphPad Prism one-way ANOVA (GraphPad Software Ver. 6, San Diego, CA, USA) was used to establish any significance differences among means of groups. The Newman–Keuls multiple comparison test was used to establish significant difference between pairs of groups.

## Results

### In vitro anti-malarial interaction assay

**Fig. 2:**
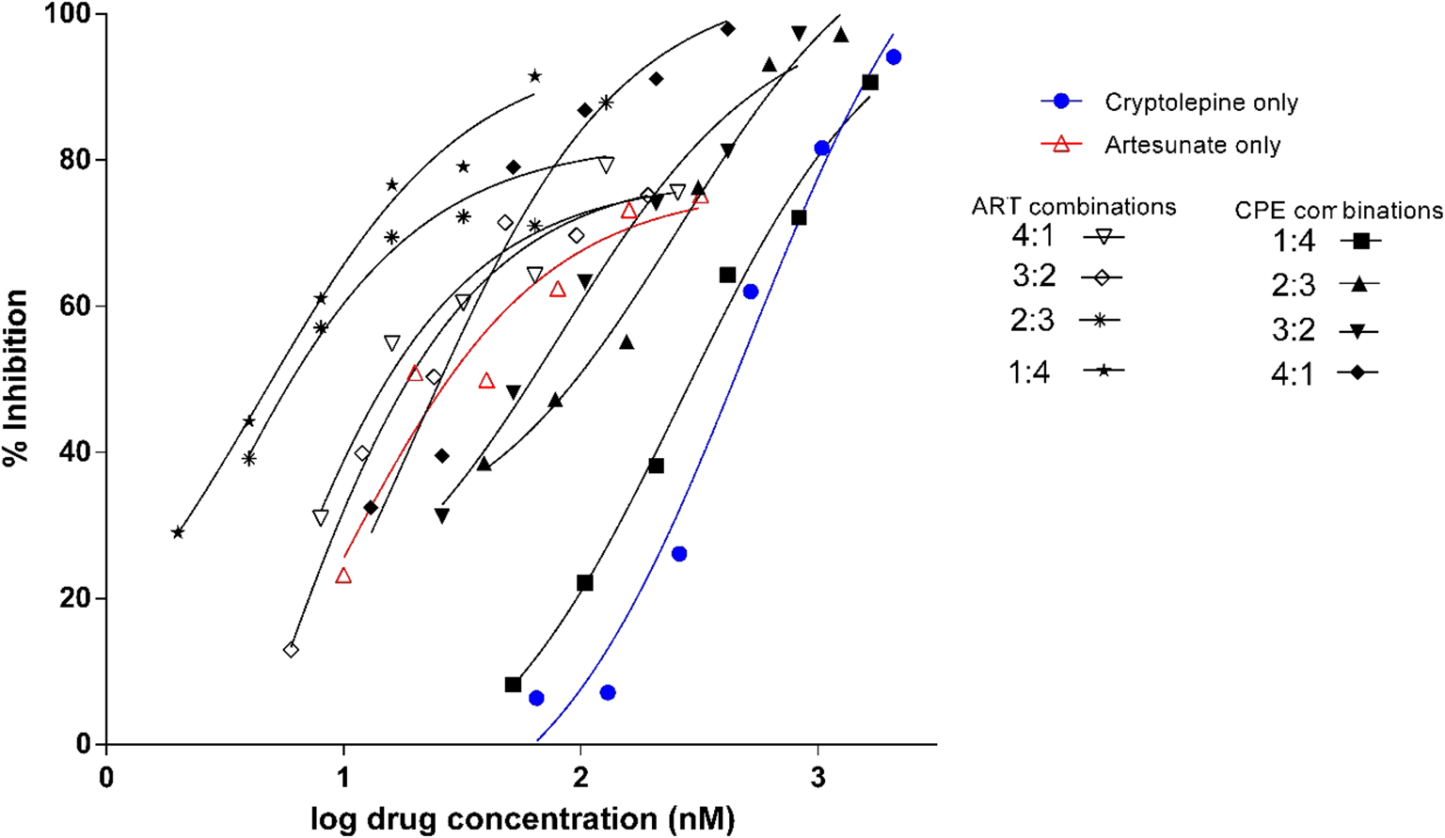
Dose-response curves from the fixed-dose combinations of cryptolepine and artesunate. Ring-stage parasites were treated with fixed-dose combinations of cryptolepine and artesunate at 48 h after which analysis was done using the SYBR Green I fluorescent method. Each value represents the IC_50_ calculated from at least three independent in vitro experiments

**Fig. 3:**
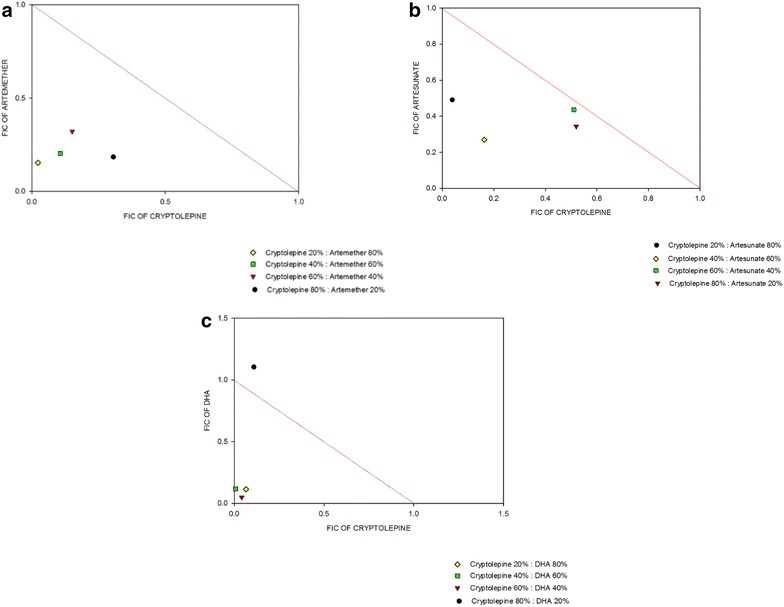
Effects of combinations of cryptolepine with the artemisinin derivatives on *Plasmodium falciparum* growth in vitro (3D7 strain). Isobolograms show the effect of combinations of both cryptolepine with artemether (**a**), artesunate (**b**) and dihydroartemisinin (**c**). The interaction between cryptolepine and artesunate, artemether, or dihydroartemisinin against ring-stage parasites were determined using the SYBR Green I fluorescent-based drug sensitivity assay using the fixed ratio method. Each combination was set up in triplicate for 48 h. The FIC_50_ concentrations were used in the plotting of the isobolograms

The stage-specific IC_50_ of CPE and the artemisinin derivatives on *P. falciparum* blood-stage cultures is shown in Table 1. In the interaction assays, the combinations of CPE with ART, ARM, and DHA were synergistic (Table 2). CPE combination with the artemisinin derivatives showed FIC_50_ of less than 0.8 suggesting synergy. The degree of synergism was stronger in ARM (ΣFIC_50_ = 0.362), followed by DHA (ΣFIC_50_ = 0.403) and finally ART (ΣFIC_50_ = 0.693) (Fig. 2). The isobolograms for the various interactions are shown in Fig. 3.

**Table 1:** IC_50_ values of cryptolepine and the artemisinin derivatives against 3D7 strain of *Plasmodium falciparum*

Drug	Mean IC_50_ ± SEM (nM)
Cryptolepine HCl	603.82 ± 75.57
Artesunate	6.76 ± 1.63
Artemether	2.59 ± 0.59
Dihydroartemisinin	6.02 ± 0.17

Values are Mean ± SEM from less than seven independent experiments

**Table 2:** Fixed-ratio interaction of drugs against *Plasmodium falciparum* 3D7 strains

Drug combination	ΣFIC_50_				Mean ΣFIC_50_	Interaction
	4:1	3:2	2:3	1:4		
Cryptolepine + artemether	0.4891	0.4727	0.3094	0.1765	0.362	Synergism
Cryptolepine + artesunate	0.8658	0.9469	0.433	0.530	0.693	Synergism
Cryptolepine + dihydroartemisinin	1.214	0.0906	0.1263	0.181	0.403	Synergism

The ratios 4:1, 3:2, 2:3 and 1:4 refer to fixed dose ratios for cryptolepine to the arteminisins. Values are expressed as the means from at least three in vitro experiments

The IC_50_ levels for the drugs alone were 6.76 ± 1.63, 2.59 ± 0.59, 6.02 ± 0.17 and 603.82 ± 75.57 nM, respectively, for ART, ARM, DHA, and CPE.

### In vivo assays

CPE produced a significant dose-dependent reduction in parasitaemia levels with similar reduction as in the ART-treated groups (positive control). The potencies of CPE and ART were 10.65 ± 0.60 mg kg^−1^ and 6.0 ± 0.05 mg kg^−1^ in the anti-malarial test (Table 3).

**Table 3:** ED_50_ of cryptolepine and artesunate in the anti-malarial test

Treatment	ED_50_ (mg kg^−1^)
Cryptolepine	10.65 ± 0.6
Artesunate	6.0 ± 0.05

Values are expressed as Mean ± SEM from less than five independent experiments

### Combination anti-malarial assay of CPE and ART

**Fig. 4:**
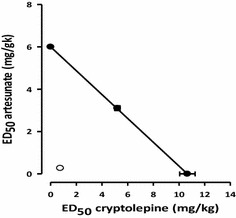
Isobologram for the combination of cryptolepine and artesunate in the Rane’s anti-malarial test. The *open circles* and *filled circles* represent the experimental and theoretical ED_50_s ± SEM, respectively

The combination of CPE (ED_50_=40 mg kg^−1^) and ART (ED_50_=6 mg kg^−1^) produced a significant reduction in parasitaemia from days 1 and 6. The combination of CPE and ART at all dose levels produced high percentage suppression in the first 3 days compared to CPE only. The lowest dose ratio combination (1/8:1/8) showed high parasite levels on days 5 and 6 compared to the negative control group (Table 4). The theoretical ED_50_s of CPE and ART combination was 8.3 ± 0.25 mg kg^−1^. The experimental ED_50_ (Zexp) of the mixture was 1.02 ± 0.02 mg kg^−1^. The Zexp (open circle) lay significantly below the line of additivity as well as the Zadd (closed circles) on the isobologram indicating synergism (Fig. 4). The degree of interaction calculated as the interaction index was 0.12 (Table 5).

**Table 4:** Percentage suppression of *P. berghei* infected mice given combination treatment

Drugs	Day 1	Day 2	Day 3	Day 4	Day 5	Day 6
Artesunate 6 mg/kg	40.74 ± 10.4	89.87 ± 2.2	97.92 ± 1.3	90.20 ± 2.1	87.40 ± 6.9	90.44 ± 2.5
Cryptolepine 40 mg/kg	53.70 ± 13.1	57.81 ± 4.0	65.3 ± 7.5	77.48 ± 15.2	82.16 ± 5.4	83.19 ± 6.4
ED_50_ (1:1)	73.15 ± 9.8	95.78 ± 9.8	93.06 ± 1.39	68.03 ± 12.6	78.35 ± 9.0	88.53 ± 4.4
ED_50_/2 (1/2:1/2)	80.56 ± 3.7	79.75 ± 5.24	88.89 ± 2.8	85.03 ± 2.7	90.81 ± 3.47	92.35 ± 2.2
ED_50_/4 (1/4:1/4)	87.96 ± 3.1	96.84 ± 1.1	76.39 ± 6.1	85.03 ± 6.8	82.94 ± 3.5	84.70 ± 2.2
ED_50_/8 (1/8:1/8)	86.11 ± 1.46	86.50 ± 2.5	77.08 ± 6.9	0.51 ± 14.65	−23.0 ± 12.4	−8.03 ± 10.3

Values are expressed as Mean ± SEM from five independent experiments

**Table 5:** Experimental (Zexp) and theoretical (Zadd) ED_50_ ± SEM values of cryptolepine and artesunate in anti-malarial test

Zexp (mg kg^−1^)	Zadd (mg kg^−1^)	Interaction index
1.02 ± 0.02***	8.3 ± 0.25	0.12

Values are expressed as Mean ± SEM

*** *P* < 0.001 compared to their respective Zadds

### Toxicological assessment of CPE and ART in healthy rats

Haematological values of treated rats were not significantly different from those of the control group for all parameters measured at doses of (25–100 mg kg^−1^) CPE except for the mean corpuscular volume (MCV) and the mean corpuscular haemoglobin (HGB) concentration (MCHC), which were significantly decreased in the group treated concomitantly with CPE (100 mg kg^−1^) and ART (4 mg kg^−1^) (Table 6).

**Table 6:** Effects of the concomitant administration of cryptolepine and artesunate 4 mg kg^−1^ on the haematological indices of Sprague-Dawley rats treated for 3 days

Parameters	Control	Cryptolepine		
		25 mg kg^−1^	50 mg kg^−1^	100 mg kg^−1^
WBC (×10^3^/µL)	9.83 ± 1.63	8.37 ± 1.54	13.03 ± 2.28	12.73 ± 1.04
RBC (×10^6^/µL)	6.41 ± 0.45	6.86 ± 0.26	6.58 ± 0.24	6.47 ± 0.29
HGB (g/dL)	11.63 ± 0.93	12.07 ± 0.20	11.77 ± 0.23	11.93 ± 0.12
HCT (%)	39.27 ± 1.68	38.4 ± 1.07	37.3 ± 0.90	35.3 ± 1.15
MCV (fL)	61.7 ± 2.19	56.03 ± 0.63	56.77 ± 0.72	54.63 ± 0.68*
MCH (pg)	18.13 ± 0.69	17.67 ± 0.44	17.9 ± 0.37	18.53 ± 0.69
MCHC (g/dL)	29.43 ± 1.15	31.47 ± 0.44	31.57 ± 0.45	33.87 ± 0.83*
PLT (×10^3^/µL)	516.33 ± 76.50	821.33 ± 9.02	725.67 ± 67.65	843.67 ± 44.67
LYMP (%)	72 ± 4.18	75.73 ± 2.80	77.38 ± 1.73	83.43 ± 2.84

Cryptolepine was combined with a fixed dose of artesunate (4 mg kg^−1^). Values are expressed as mean ± SEM (n=5), compared to the controls (ANOVA) followed by Student‘s Newman–Keuls multiple comparison test

*Asterisk* indicates significance (*P* < 0.05)

The oral administration of CPE only as well as with ART did not cause any significant changes in serum proteins, bilirubin, liver enzymes, creatinine, urea, and uric acid.

### Haematological analysis of *Plasmodium berghei*-infected mice treated with various concentrations of CPE and ART

**Fig. 5:**
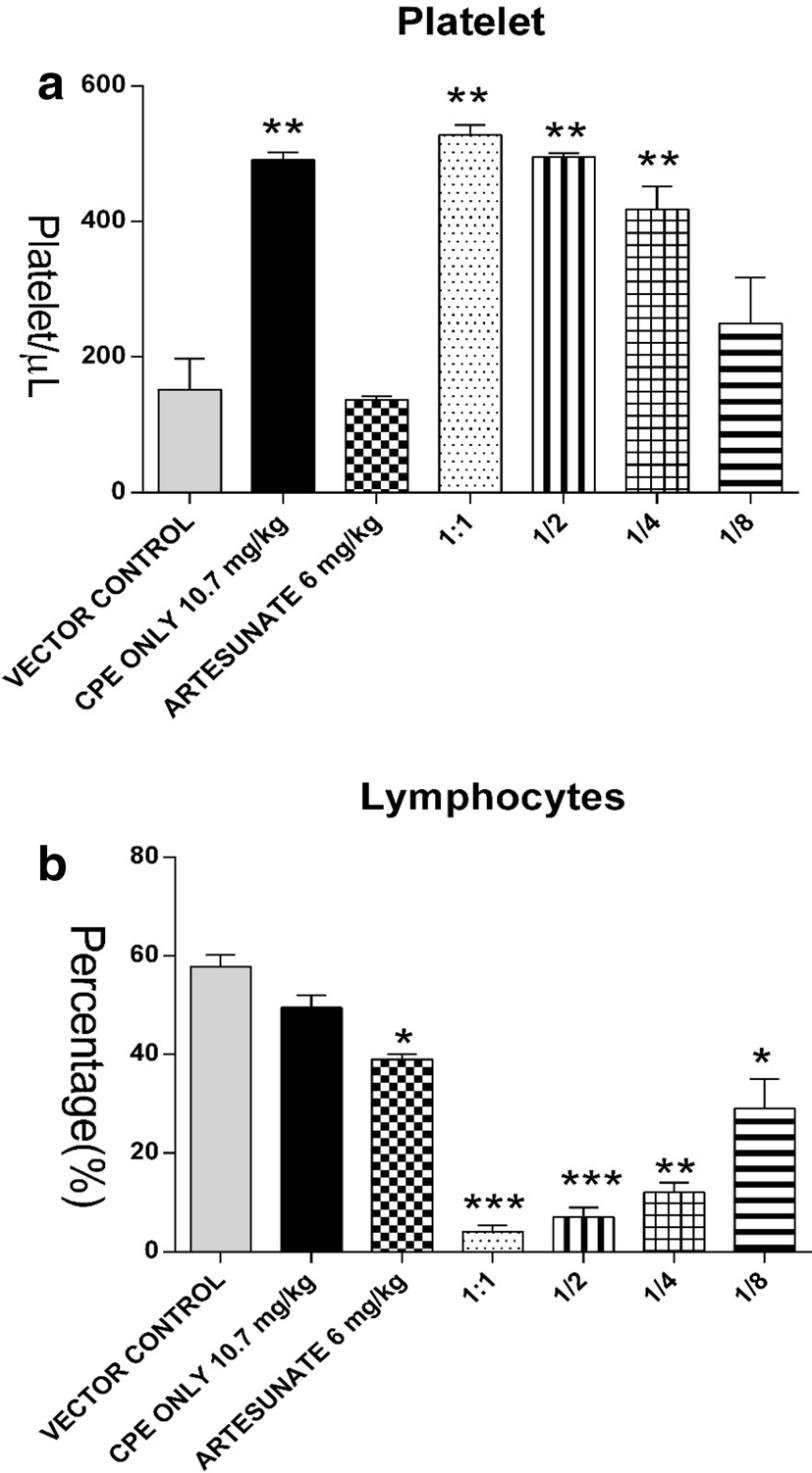
Effect of cryptolepine and artesunate on the haematological indices (**a** Platelets **b** Lymphocytes) of *Plasmodium berghei*-infected ICR mice treated for 6 days. Values are expressed as Mean ± SEM (n=5), *Asterisk* indicates significance (P < 0.05), *double Asterisk* indicates significance (P < 0.01) and *triple Asterisk* indicates significance (P < 0.001) compared to controls (ANOVA) followed by Student’s Newman–Keuls multiple comparison test

Figure 5 shows the haematological parameters after 5 days of treatment. A significant increase in platelet (PLT) count was observed in all groups treated with CPE compared to the vector control. Lymphocyte (LYMP) levels were generally lowered in all groups compared to the vector control.

### Histopathology

**Fig. 6:**
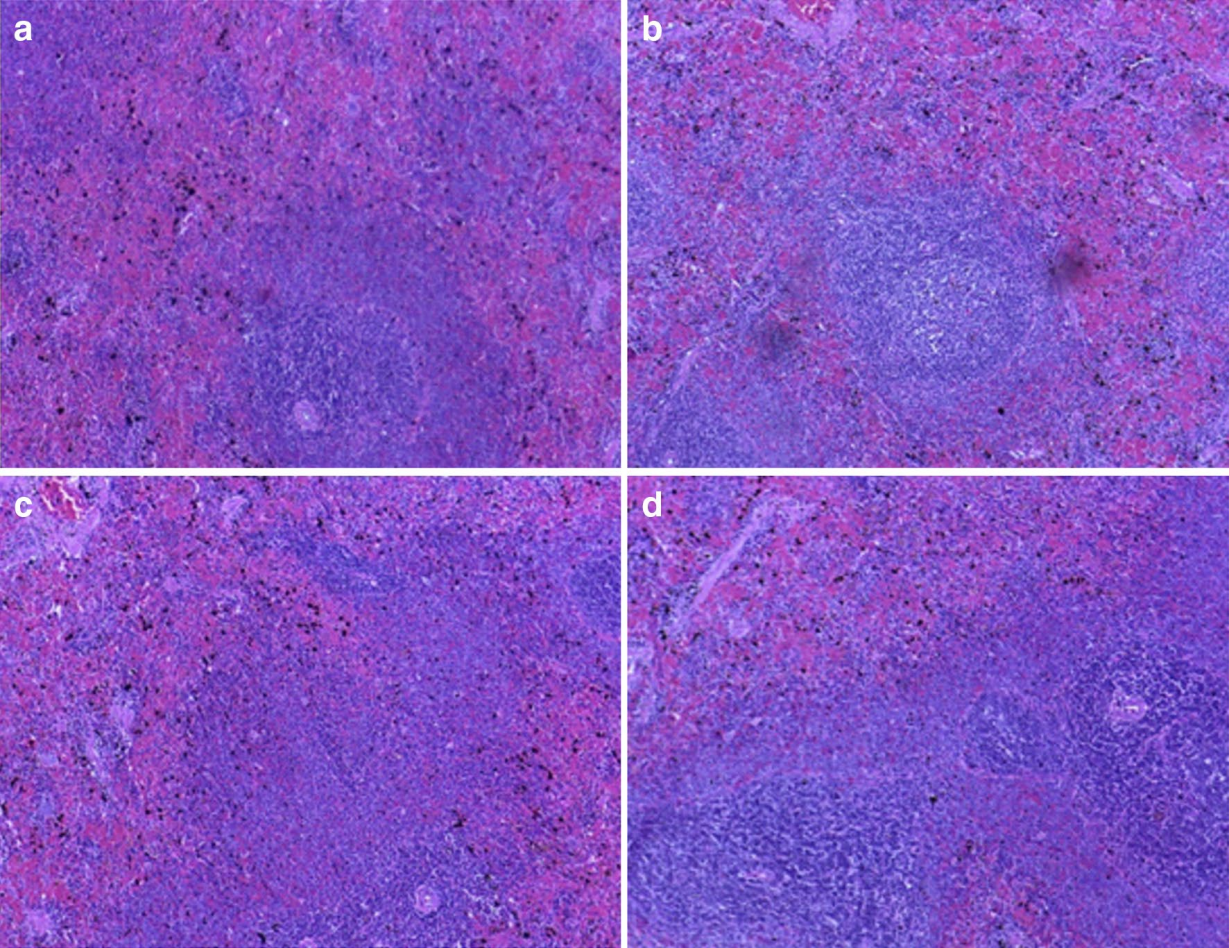
Photomicrograph (×100) showing histopathological slides of the spleen of cryptolepine (CPE) and/artesunate (ART, 6 mg kg^−1^) treated rats. **a** Control **b** Artesunate 6 mg kg^−1^ **c** CPE 100 mg kg^−1^ **d** CPE 100 mg kg^−1^ + ART 6 mg kg^−1^, all treated for 3 days

**Fig. 7:**
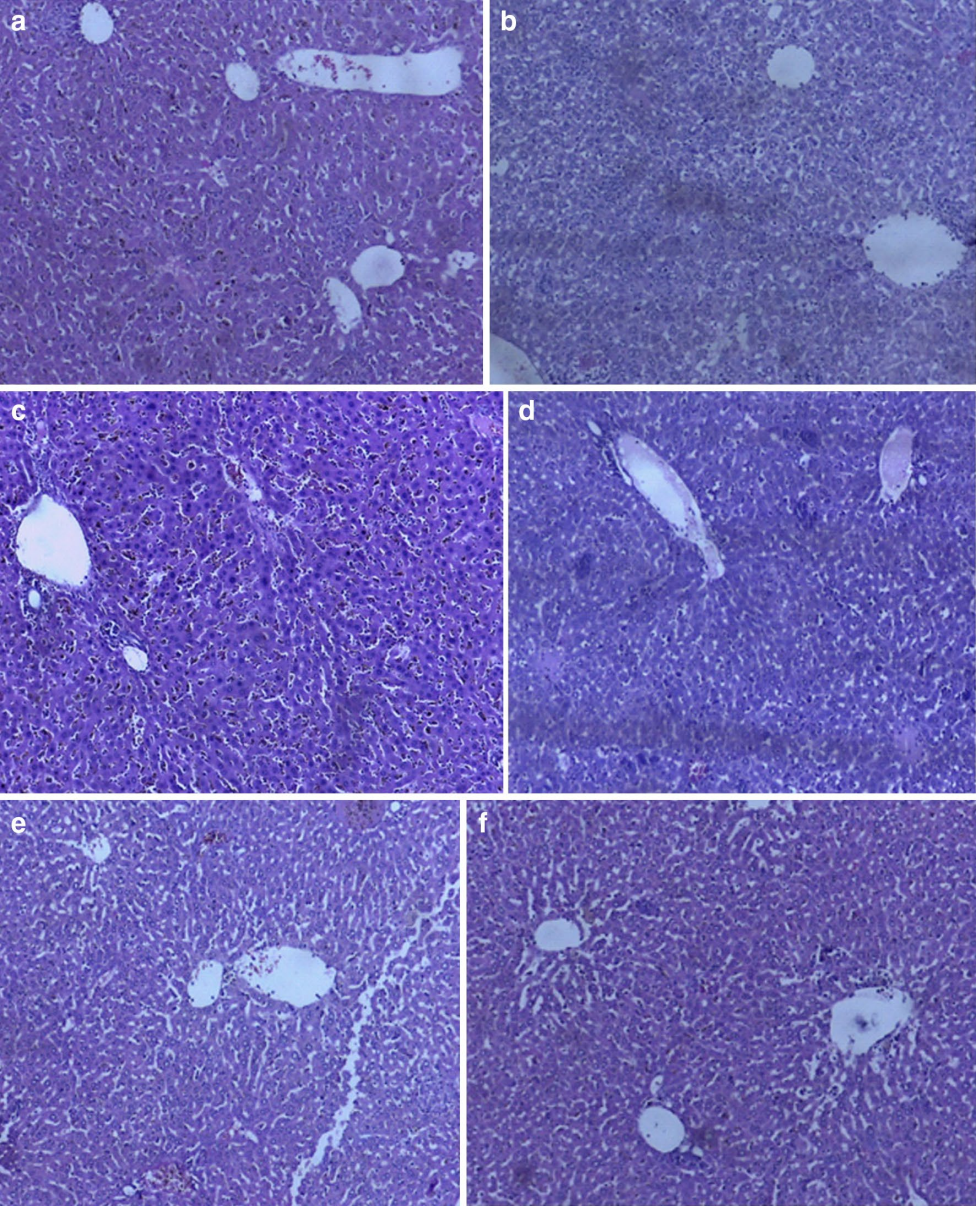
Photomicrograph (×100) showing histopathological slides of the livers of cryptolepine (CPE) and/or artesunate (ART, 6 mg kg^−1^) treated *Plasmodium berghei*-infected mice. **a** Control, **b** ED_50_ of ART (6 mg kg^−1^), **c** ED_50_ of CPE (10.7 mg kg^−1^) **d** ED_50_ (CPE + ART) **e** ½ ED_50_ (CPE + ART) **f** ¼ ED_50_ (CPE + ART)

The livers, kidneys, spleens, and stomachs from the distilled water-treated (control) group had normal appearance and histology. Generally, no observable changes in the architecture of these organs of treated animals compared to the control (Figs. 6, 7). The histology of the liver and kidney were consistent with the normal alanine transaminase, alkaline phosphate, bilirubin, creatinine, and urea levels observed in the serum.

## Discussion

The discovery of artemisinin and its derivatives from the leafy portions of *Artemisia annua* has been the major advance in the chemotherapy of malaria [25]. ACT is now the mainstay in the treatment of uncomplicated malaria due to the high efficacy and low probability of drug-resistance development [26].

The present study focuses on the combination of the plant-derived anti-malarial compound, CPE, with the widely used artemisinin derivatives, with the aim of developing novel artemisinin combinations to forestall the progression of resistance to these agents. Several reports on the antiplasmodial activities of extracts of *C. sanguinolenta* and its major alkaloid, CPE, have been extensively studied in vivo and in vitro [11, 12]. In this study, the interactions observed when this indoloquinoline is combined with some artemisinin derivatives both in vitro and in vivo were demonstrated. The susceptibilities of CPE and the standard drugs in vitro were close to those reported in literature [27, 28]. The in vitro anti-malarial interactions of the different combinations are shown in the isobologram analysis in Figs. 3 and 4. CPE in combination with ART, ARM and DHA had the ΣFIC always below the line of additivity. The values obtained with these three artemisinins indicate a synergistic interaction of the artemisinins with CPE. The mechanism of action of the indoloquinolones has been shown to be similar to that of CQ in inhibiting the conversion of poisonous haem to haemozoin (β-haematin) in the parasite food vacuole [29].

In the in vivo assay, all the combination treatment produced a more significant reduction in parasitaemia compared to the use of only CPE or ART on the first day of treatment. This translated into a synergistic effect when the two agents were used together. The rapid onset of anti-malarial activity continued through to the first 3 days of the combination treatment at all dose ratios. This indicates a possible rapid onset of antiplasmodial activity when CPE is used in combination with ART compared to each of the drugs used alone. With the current 3 day anti-malarial treatment, a combination of CPE with ART may offer better choice for rapid clearance of parasites in the blood compared to any of the two agents used alone. Again, the long duration of action of this combination will ensure efficient parasite clearance. The mechanisms involved in the enhanced activity observed with CPE-artemisinin combinations have not yet been elucidated. However, concerning the synergistic effect observed with the artemisinins and amodiaquine, further studies should be performed with CPE alone and in combination on several *P. falciparum* strains and/or rodent malaria models to highlight the biochemical mechanisms behind its antiplasmodial interactions. On this basis, it is strongly believed that the combination of CPE with the artemisinin derivatives is a legitimate choice for an alternative anti-malarial combination development.

The haematological parameters showed no significant difference in the total white blood cell count in all treated groups compared to the vector control, except the group treated with the combination of the ED_50_ of CPE and ART (1:1 ratio). The LYMP levels were significantly decreased in all combination treatments. Generally, malaria infections are usually associated with low levels of LYMPs indicative of a compromised immune response [30]. In a clinical trial using tea bags containing *C. sanguinolenta*, a progressive increase in PLT count after treatment was observed in the human subjects used. The high PLT count observed was concluded as an indication of the effectiveness of the tea bag against *falciparum* malaria [31]. In the current study, the PLT count in all CPE-treated groups was significantly increased (P <0.001) compared to the vector control. This may be an inherent attempt to boost the acute thrombocytopaenia and immune suppression usually accompanied with malaria [32]. PLTs assist and modulate inflammatory reactions and immune responses. The lowest dose ratio of CPE with ART did not show any significant change in the PLT level compared to the vector control.

The administration of *C. sanguinolenta* to rats has been shown to cause hyperplasia and hypertrophy of gastric parietal cells [33]. In this study, the histology of the stomach of mice treated with CPE and the various combinations were examined. No observable defects were observed in the architecture of the stomach of the mice. The liver architecture showed no deformity in the drug-treated group compared to the control. This, coupled with no reported deaths in the 9-day treatment shows the safety of the combination of CPE with ART in the acute treatment in mice.

The safety evaluation of CPE when used with the ART was further determined in healthy Sprague-Dawley rats. The analysis of the cellular component of blood is pertinent to risk assessment as the haematological system provides predictive indices for toxicity in mammals [34]. CPE at all dose levels showed no significant detrimental effect in the haematological parameters evaluated compared to the control group for the 3-day period. The concurrent use of CPE did not cause any significant change in the haemoglobin (Hb) concentration, therefore the reduced MCV reflected in a rise in MCHC, a measure of the average concentration of haemoglobin in a RBC. The RBCs in this case appeared microcytic however; the haemoglobin levels in cells were not affected.

The levels of cellular enzymes in the serum and other body fluids play an important role in the diagnosis of tissue and organ injury [35]. Aspartate and alanine transaminases are useful marker enzymes in assessing liver damage [36] and their detection in the serum is indicative of possible liver dysfunction [37]. However, they are not always good indications of how well the liver is functioning as elevation of these enzymes are often unexpectedly encountered on routine blood screening test in otherwise healthy individuals [38]. CPE with or without ART showed no significant difference in the biochemical parameters compared to the control group. Microscopic observations revealed a normal hepatocyte architecture with a well-defined central vein. No necrosis, steatosis, chronic inflammatory infiltration, or degenerative changes were observed in any of the drug-treated animals. Gross morphological inspection of other organs (kidney, spleen and stomach) also revealed no apparent damage. The results were consistent with the liver and kidney function test, which showed values not significantly different from the controls used. In the *P. berghei*-infected mice, histopathology of the liver, spleen, stomach, and kidney in all treatment groups were not different from the control groups. Despite these reports of safety in the acute toxicity studies in rodents, CPE has been reported to be a DNA intercalator and also possess genotoxic properties in mammalian cells [39]. Though this study did not focus on possible chronic toxicity, anti-malarial combination therapies are mostly taken over a 3-day period. Potential chronic toxicity from such combinations can therefore be precluded. Generally, therefore combination therapy of CPE–ART demonstrated in the present study appears safe.

## Conclusion

The combination of CPE with the artemisinins showed a synergistic effect both in vivo and in vitro against *P. falciparum* 3D7 and *P.berghei* NK-65, respectively. The combination of CPE with ART did not cause acute toxicity as no significant changes in histopathology, biochemical and the haematological parameters were observed in healthy Sprague-Dawley rats.

## Abbreviations

FIC: fractional inhibitory concentrations
Mean ΣFICs: mean sums of fractional inhibitory concentrations
IC_50_: 50 % inhibitory concentration
WHO: World Health Organization
ED_50_: 50 % effective dose
RBC: red blood cell
ACT: artemisinin-based combination therapy
ART: artesunate
ARM: artemether
CPE: cryptolepine
DHA: dihydroartemisinin
CQ: chloroquine
nM: nano-molar
mM: milli-molar
CPM: complete parasite medium
MSF: malaria SYBR Green I fluorescence
MCV: mean corpuscular volume
MCHC: mean corpuscular haemoglobin concentration
WBC: white blood cell
HGB: haemoglobin
PLT: platelet
LYMP: lymphocyte
EDTA: ethylene diaminetetraacetic acid
