# Erratum to: Synergistic anti-malarial action of cryptolepine and artemisinins

**DOI:** 10.1186/s12936-016-1223-8

**Published:** 2016-03-16

**Authors:** Arnold D. Forkuo, Charles Ansah, Kwesi M. Boadu, Johnson N. Boampong, Elvis O. Ameyaw, Ben A. Gyan, Andrea T. Arku, Michael F. Ofori

**Affiliations:** Department of Pharmacology, Faculty of Pharmacy and Pharmaceutical Sciences, College of Health Sciences, Kwame Nkrumah University of Science and Technology, Kumasi, Ghana; Department of Biomedical and Forensic Sciences, School of Biological Science, College of Agriculture and Natural Sciences, University of Cape Coast, Cape Coast, Ghana; Department of Immunology, Noguchi Memorial Institute for Biomedical Research, University of Ghana, Legon, Ghana

## Erratum to: Malar J (2016) 15:89 DOI 10.1186/s12936-016-1137-5

After publication of the original article [1], the authors found that there was an error in the first sentence of the ‘Conclusions’ section: “The combination of CPE with the artemisinins showed a synergistic effect both in vivo and in vitro against *P. falciparum* 3D7 and *P. berghei* NK-65, respectively.”

Within this sentence, the appearance of ‘*P. falciparum* 3D7’ and ‘*P. berghei* NK-65’ should have been reversed. The sentence in the original article has been corrected to read as follows: “The combination of CPE with the artemisinins showed a synergistic effect both in vivo and in vitro against *P. berghei* NK-65 and *P. falciparum* 3D7, respectively.”
